# Crystal structure of pirfenidone (5-methyl-1-phenyl-1*H*-pyridin-2-one): an active pharmaceutical ingredient (API)

**DOI:** 10.1107/S2056989019006418

**Published:** 2019-06-11

**Authors:** Mauro Barbero, Matteo Mossotti, Angelo Sironi, Giovanni Battista Giovenzana, Valentina Colombo

**Affiliations:** a Dipartimento di Scienze del Farmaco, Università del Piemonte Orientale, Largo Donegani 2/3, I-28100, Novara, Italy; b R&D Division, PROCOS S.p.A., Via G. Matteotti 249, 28062 Cameri (Novara), Italy; c Dipartimento di Chimica, Università degli Studi di Milano, Via Golgi 19, I-20133 Milano, Italy

**Keywords:** crystal structure, pirfenidone, active pharmaceutical ingredient (API), idiopathic pulmonary fibrosis (IPF), hydrogen bonding

## Abstract

The crystal structure of pirfenidone, C_12_H_11_NO [alternative name: 5-methyl-1-phenyl­pyridin-2(1*H*)-one], an active pharmaceutical ingredient (API) approved in Europe and Japan for the treatment of Idiopathic pulmonary fibrosis (IPF), is reported here for the first time. It was crystallized from toluene by the temperature gradient technique, and crystallizes in the chiral monoclinic space group *P*2_1_. The phenyl and pyridone rings are inclined to each other by 50.30 (11)°. In the crystal, mol­ecules are linked by C–H⋯O hydrogen bonds involving the same acceptor atom, forming undulating layers lying parallel to the *ab* plane.

## Abstract 2

The crystal structure of pirfenidone, [5-methyl-1-phenyl­pyridin-2(1*H*)-one], an active pharmaceutical ingredient (API) approved in Europe and Japan for the treatment of idiopathic pulmonary fibrosis (IPF), is reported here for the first time. It was crystallized from toluene by the temperature gradient technique, and crystallizes in the chiral monoclinic space group *P*2_1_.

## Chemical context   

Idiopathic Pulmonary Fibrosis (IPF) is a lung disease characterized by cough, scars and dyspnea that leads to progressive and irreversible loss of lung function. Pirfenidone (systematic name: 5-methyl-1-phenyl-1*H*-pyridin-2-one) has been approved in Japan since 2008 (Pirespa^®^) and in Europe since 2011 (Esbriet^®^) for the treatment of IPF, even if its mechanism of action has not been completely elucidated (Richeldi *et al.*, 2011 ▸). Different synthetic approaches have been reported, mainly relying on *N*-aryl­ation reactions of 5-methyl-2-pyridone (Liu *et al.*, 2009 ▸; Crifar *et al.*, 2014 ▸; Jung *et al.*, 2016 ▸; Falb *et al.*, 2017 ▸). Pirfenidone has been known since 1974 (Gadekar, 1974 ▸) and its anti­fibrotic properties were described in 1990 (Margolin, 1990 ▸). Nevertheless, despite its formulation as oral tablets, no information on the solid-state structure of this compound has been reported to date. In the present study, we report and analyse the crystal structure of pirfenidone.
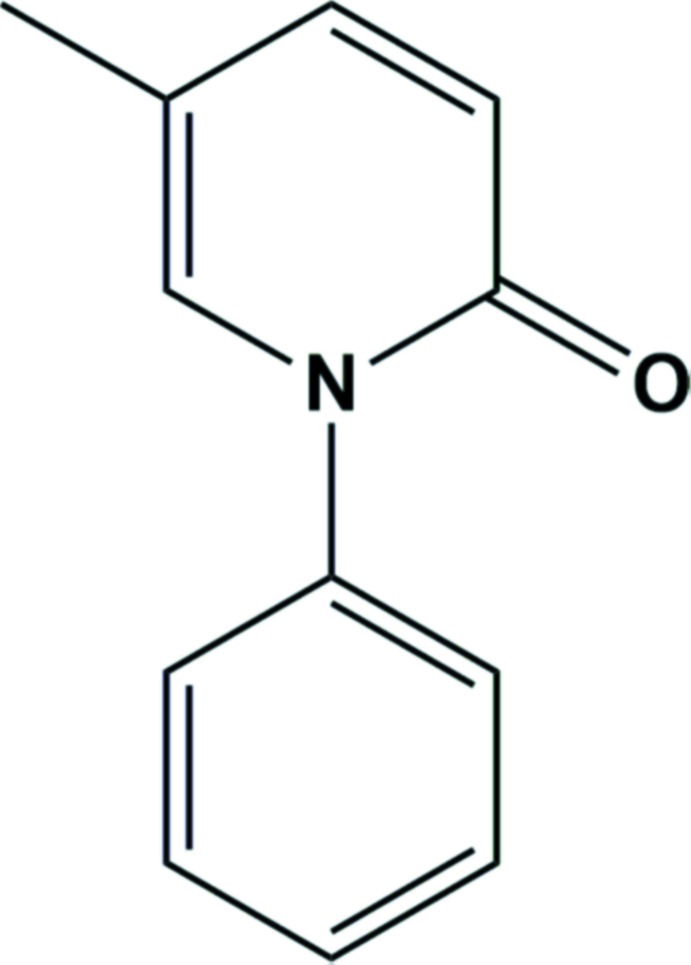


## Structural commentary   

**Figure 1:**
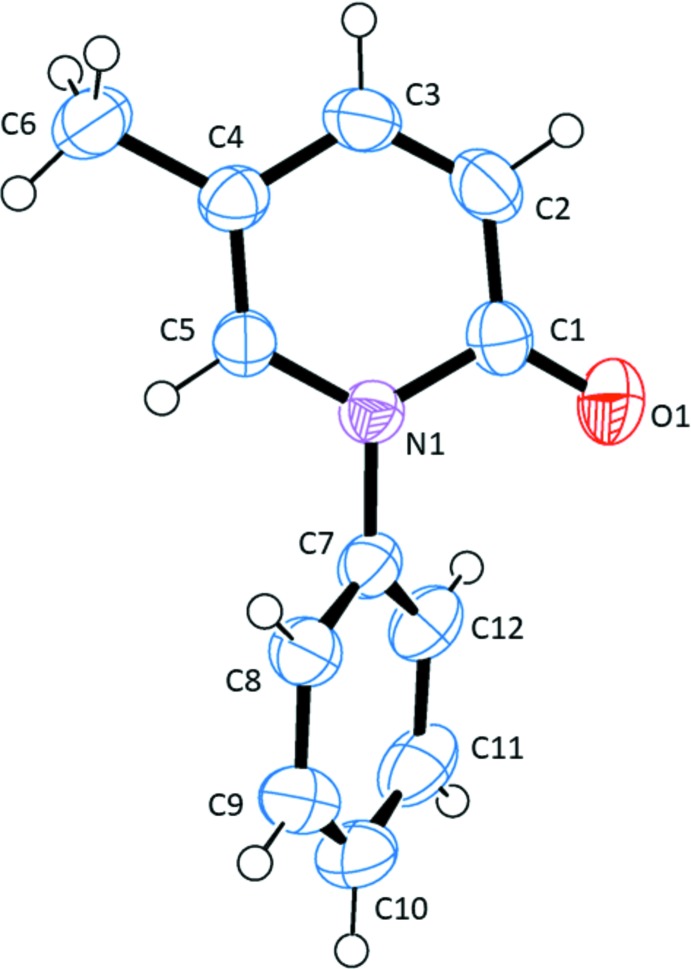
A view of the mol­ecular structure of pirfenidone with the atom labelling. Displacement ellipsoids are drawn at the 50% probability level.

The mol­ecular structure of pirfenidone is shown in Fig. 1 ▸. This axially chiral mol­ecule crystallizes in the monoclinic space group *P*2_1_, with one mol­ecule in a general position. The mol­ecule is far from planar with the phenyl (C7–C12) and pyridinone (N1/C1–C5) rings subtending a dihedral angle of 50.30 (11)°.

## Supra­molecular features   

**Figure 2:**
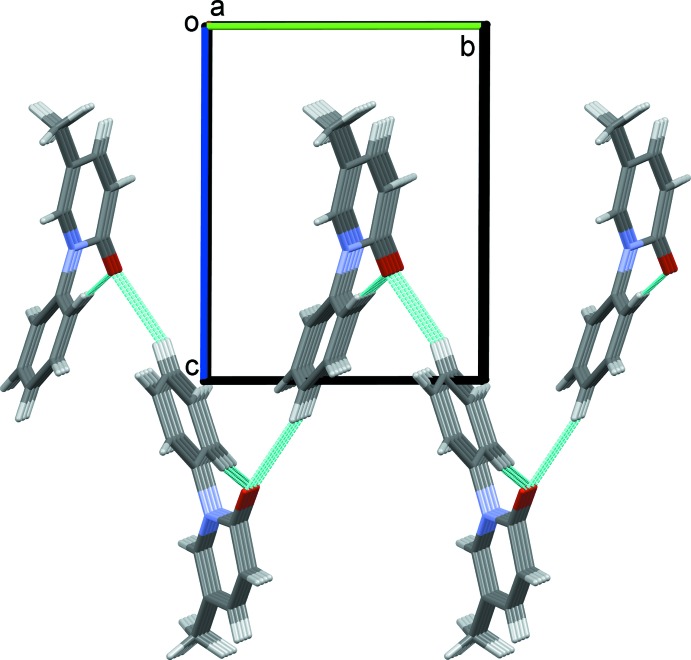
A view along the *a* axis of the crystal packing of pirfenidone. The C—H⋯O hydrogen bonds (see Table 1 ▸) are shown as dashed lines.

**Figure 3:**
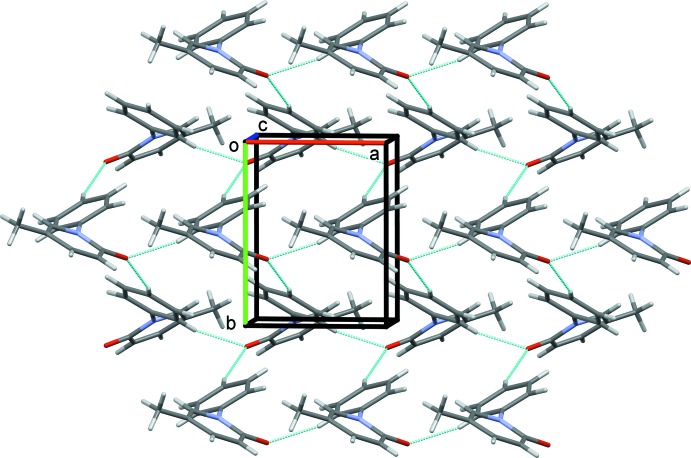
A view along the *c* axis of the crystal packing of pirfenidone. The C—H⋯O hydrogen bonds (see Table 1 ▸) are shown as dashed lines.

In the crystal, mol­ecules are linked by C—H⋯O hydrogen bonds involving the same acceptor atom (Table 1 ▸), forming an undulating network, enclosing 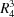
(20) ring motifs, and lying parallel to the *ab* plane (Figs. 2 ▸ and 3 ▸). The 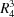
(20) ring motifs are clearly visible in Fig. 3 ▸. There are no other significant inter­molecular contacts present according to the analysis of the crystal structure using *PLATON* (Spek, 2009 ▸).

## Database Survey   

A search of the Cambridge Structural Database (CSD, Version 5.40, February 2019; Groom *et al.*, 2016 ▸) for 1-phenyl­pyridin-2(1*H*)-ones, excluding structures with ring atoms being included in further cyclic moieties, gave 40 hits (see supporting information file S1). Only six of these compounds involve an unsubstituted phenyl ring as in the title compound. When considering compounds with no substituent in position-6 of the pyridinone ring (on atom C5 in the title compound; Fig. 1 ▸) only three structures fit this extra criteria, *viz. S*-ethyl 2-oxo-1-phenyl-1,2-di­hydro-3-pyridine­carbo­thio­ate (CSD refcode NOLBIA; Liu *et al.*, 2008 ▸), monoclinic space group *P*2_1_, 4-chloro-6-oxo-1-phenyl-1,6-di­hydro­pyridine-3-carbaldehyde (QIWFIM; Xiang *et al.*, 2008 ▸), monoclinic space group *P*2_1_/*c*, and methyl 5-benzoyl-2-oxo-1-phenyl-1,2-di­hydro­pyridine-4-carboxyl­ate (TEMKIH; Shao *et al.*, 2012 ▸), ortho­rhom­bic space group *Pna*2_1_ with two independent mol­ecules in the asymmetric unit. In these three compounds, the phenyl ring is inclined to the pyridone ring by *ca* 65.50, 64.66 and 55.83/57.12°, respectively. This dihedral angle in the title compound, pirfenidone, is 50.30 (11)°. In the other three compounds [AQIKIV (Gorobets *et al.*, 2010 ▸), BAFPUV (Dyachenko *et al.*, 2011 ▸) and WEDCEP (Allais *et al.*, 2012 ▸) – see supporting information file S1] with a substituent in position-6 of the pyridinone ring the corresponding dihedral angle varies from *ca* 73.02 to 89.28° as a result of steric hindrance.

## Synthesis and crystallization   

Pirfenidone was obtained in > 99.5% purity according to the method published previously (Mossotti *et al.*, 2018 ▸). Single crystals were grown in the following way: approximately 100 mg of pirfenidone in 2 mL of toluene was heated until complete dissolution. The flask with this solution was then closed and kept at 273–278 K. Well-formed colourless crystals of pirfenidone were obtained after 1 week. The melting point of this crystal form, determined by DSC analysis (heating rate 10 K min^−1^), is 383 K. This crystallization procedure must be performed in order to grow single crystals suitable for X-ray diffraction analysis and not with the aim of increasing the purity of the product. It is worth nothing that the industrial process is already optimized for the isolation of a pure API (> 99.5%) and a further crystallization step is not needed to improve its purity. We performed several other crystallization trials in order to search for other possible forms of pirfenidone; however, each crystallization attempt gave rise to the same crystal form.

## Refinement   

Crystal data, data collection and structure refinement details are summarized in Table 2 ▸. The H atoms were included in calculated positions and treated as riding: C—H = 0.93–0.96 Å with *U*_iso_(H) = 1.5*U*_eq_(C-meth­yl) and 1.2*U*_eq_(C) for other H atoms.

## Supplementary Material

Crystal structure: contains datablock(s) Pirfenidone, Global. DOI: 10.1107/S2056989019006418/tx2011sup1.cif

CSD search results. DOI: 10.1107/S2056989019006418/tx2011sup3.pdf

CCDC reference: 1914224

Additional supporting information:  crystallographic information; 3D view; checkCIF report

## Figures and Tables

**Table 1:** Hydrogen-bond geometry (Å, °)

*D*—H⋯*A*	*D*—H	H⋯*A*	*D*⋯*A*	*D*—H⋯*A*
C8—H8⋯O1^i^	0.93	2.33	3.203 (3)	156
C10—H10⋯O1^ii^	0.93	2.46	3.310 (3)	152

Symmetry codes: (i) 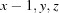; (ii) 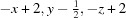.

**Table 2:** Experimental details

Crystal data	
Chemical formula	C_12_H_11_NO
*M*_r_	185.22
Crystal system, space group	Monoclinic, *P*2_1_
Temperature (K)	293
*a*, *b*, *c* (Å)	6.2525 (8), 7.797 (1), 10.2810 (13)
β (°)	104.744 (2)
*V* (Å^3^)	484.70 (11)
*Z*	2
Radiation type	Mo *K*α
μ (mm^−1^)	0.08
Crystal size (mm)	0.50 × 0.45 × 0.05

Data collection	
Diffractometer	Bruker SMART APEX CCD
Absorption correction	Multi-scan (*SADABS*; Bruker, 2010 ▸)
*T*_min_, *T*_max_	0.692, 0.746
No. of measured, independent and observed [*I* > 2σ(*I*)] reflections	4547, 2128, 1879
*R*_int_	0.019
(sin θ/λ)_max_ (Å^−1^)	0.643

Refinement	
*R*[*F* ^2^ > 2σ(*F* ^2^)], *wR*(*F* ^2^), *S*	0.037, 0.095, 1.04
No. of reflections	2128
No. of parameters	127
No. of restraints	1
H-atom treatment	H-atom parameters constrained
Δρ_max_, Δρ_min_ (e Å^−3^)	0.11, −0.20
Absolute structure	Flack *x* determined using 762 quotients [(*I* ^+^)−(*I* ^−^)]/[(*I* ^+^)+(*I* ^−^)] (Parsons *et al.*, 2013 ▸)
Absolute structure parameter	0.3 (4)

Computer programs: *APEX2* and *SAINT* (Bruker, 2010 ▸), *SHELXT2017* (Sheldrick, 2015*a* ▸), *SHELXL2017* (Sheldrick, 2015*b* ▸), *ORTEP-3 for Windows* (Farrugia, 2012 ▸), *Mercury* (Macrae *et al.*, 2008 ▸), *PLATON* (Spek, 2009 ▸) and *publCIF* (Westrip, 2010 ▸).

## supplementary crystallographic information

### Crystal data

**Table:**

C_12_H_11_NO	*D*_x_ = 1.269 Mg m^−^^3^
*M**_r_* = 185.22	Melting point: 375 K
Monoclinic, *P*2_1_	Mo *K*α radiation, λ = 0.71073 Å
*a* = 6.2525 (8) Å	Cell parameters from 2547 reflections
*b* = 7.797 (1) Å	θ = 3.3–27.2°
*c* = 10.2810 (13) Å	µ = 0.08 mm^−^^1^
β = 104.744 (2)°	*T* = 293 K
*V* = 484.70 (11) Å^3^	Plate, colourless
*Z* = 2	0.50 × 0.45 × 0.05 mm
*F*(000) = 196	

### Data collection

**Table:**

Bruker SMART APEX CCD diffractometer	1879 reflections with *I* > 2σ(*I*)
ω scans	*R*_int_ = 0.019
Absorption correction: multi-scan (SADABS; Bruker, 2010)	θ_max_ = 27.2°, θ_min_ = 2.1°
*T*_min_ = 0.692, *T*_max_ = 0.746	*h* = −8→8
4547 measured reflections	*k* = −9→10
2128 independent reflections	*l* = −13→13

### Refinement

**Table:**

Refinement on *F*^2^	Hydrogen site location: inferred from neighbouring sites
Least-squares matrix: full	H-atom parameters constrained
*R*[*F*^2^ > 2σ(*F*^2^)] = 0.037	*w* = 1/[σ^2^(*F*_o_^2^) + (0.0528*P*)^2^ + 0.0476*P*] where *P* = (*F*_o_^2^ + 2*F*_c_^2^)/3
*wR*(*F*^2^) = 0.095	(Δ/σ)_max_ < 0.001
*S* = 1.04	Δρ_max_ = 0.11 e Å^−^^3^
2128 reflections	Δρ_min_ = −0.20 e Å^−^^3^
127 parameters	Absolute structure: Flack *x* determined using 762 quotients [(*I*^+^)-(*I*^-^)]/[(*I*^+^)+(*I*^-^)] (Parsons *et al.*, 2013)
1 restraint	Absolute structure parameter: 0.3 (4)

### Special details

**Table:**

Geometry. All esds (except the esd in the dihedral angle between two l.s. planes) are estimated using the full covariance matrix. The cell esds are taken into account individually in the estimation of esds in distances, angles and torsion angles; correlations between esds in cell parameters are only used when they are defined by crystal symmetry. An approximate (isotropic) treatment of cell esds is used for estimating esds involving l.s. planes.
Refinement. none

### Fractional atomic coordinates and isotropic or equivalent isotropic displacement parameters (Å^2^)

**Table:**

	*x*	*y*	*z*	*U*_iso_*/*U*_eq_	
N1	0.7599 (2)	0.5275 (2)	0.62509 (16)	0.0382 (4)	
O1	1.0951 (2)	0.6634 (3)	0.69330 (17)	0.0602 (5)	
C1	0.9386 (3)	0.6188 (3)	0.6002 (2)	0.0437 (5)	
C5	0.5769 (3)	0.4849 (3)	0.5233 (2)	0.0395 (5)	
H5	0.460421	0.428046	0.545647	0.047*	
C7	0.7635 (3)	0.4782 (3)	0.76141 (19)	0.0401 (5)	
C4	0.5595 (3)	0.5219 (3)	0.3929 (2)	0.0430 (5)	
C12	0.9445 (4)	0.3913 (3)	0.8390 (2)	0.0497 (5)	
H12	1.066616	0.368712	0.805687	0.060*	
C8	0.5830 (3)	0.5134 (3)	0.8108 (2)	0.0481 (5)	
H8	0.462114	0.572084	0.758231	0.058*	
C6	0.3599 (4)	0.4732 (3)	0.2835 (2)	0.0579 (6)	
H6A	0.400128	0.387266	0.227127	0.087*	
H6B	0.304346	0.572565	0.230447	0.087*	
H6C	0.247695	0.428652	0.322817	0.087*	
C11	0.9409 (5)	0.3383 (3)	0.9672 (2)	0.0616 (7)	
H11	1.061057	0.278817	1.019737	0.074*	
C3	0.7416 (4)	0.6079 (3)	0.3637 (2)	0.0527 (6)	
H3	0.737708	0.633055	0.274701	0.063*	
C2	0.9189 (4)	0.6537 (3)	0.4608 (2)	0.0540 (6)	
H2	1.033979	0.710617	0.436893	0.065*	
C9	0.5836 (4)	0.4604 (4)	0.9395 (2)	0.0623 (7)	
H9	0.462501	0.484254	0.973553	0.075*	
C10	0.7616 (5)	0.3728 (4)	1.0174 (2)	0.0649 (7)	
H10	0.760634	0.337171	1.103577	0.078*	

### Atomic displacement parameters (Å^2^)

**Table:**

	*U*^11^	*U*^22^	*U*^33^	*U*^12^	*U*^13^	*U*^23^
N1	0.0355 (8)	0.0426 (9)	0.0362 (8)	0.0009 (8)	0.0084 (7)	0.0007 (7)
O1	0.0399 (8)	0.0725 (11)	0.0631 (11)	−0.0111 (8)	0.0038 (7)	−0.0046 (9)
C1	0.0368 (10)	0.0430 (12)	0.0524 (12)	0.0007 (9)	0.0134 (9)	−0.0023 (10)
C5	0.0380 (9)	0.0395 (11)	0.0398 (10)	−0.0026 (9)	0.0078 (8)	0.0011 (9)
C7	0.0411 (10)	0.0397 (11)	0.0362 (10)	−0.0013 (8)	0.0034 (8)	0.0002 (8)
C4	0.0524 (11)	0.0376 (11)	0.0376 (10)	−0.0009 (10)	0.0087 (9)	−0.0001 (9)
C12	0.0496 (12)	0.0478 (12)	0.0459 (12)	0.0081 (10)	0.0017 (10)	−0.0051 (10)
C8	0.0395 (10)	0.0602 (13)	0.0435 (11)	−0.0003 (10)	0.0082 (8)	0.0070 (11)
C6	0.0705 (15)	0.0570 (15)	0.0399 (11)	−0.0077 (13)	0.0026 (11)	−0.0004 (11)
C11	0.0708 (16)	0.0522 (14)	0.0469 (13)	0.0053 (12)	−0.0126 (12)	0.0046 (11)
C3	0.0694 (15)	0.0522 (14)	0.0410 (11)	−0.0091 (11)	0.0224 (11)	0.0002 (10)
C2	0.0559 (13)	0.0556 (14)	0.0576 (14)	−0.0113 (12)	0.0276 (11)	−0.0015 (11)
C9	0.0595 (14)	0.0851 (19)	0.0450 (13)	−0.0110 (13)	0.0183 (11)	0.0036 (13)
C10	0.0826 (19)	0.0688 (17)	0.0378 (12)	−0.0134 (16)	0.0054 (12)	0.0103 (12)

### Geometric parameters (Å, º)

**Table:**

N1—C5	1.381 (2)	C8—C9	1.386 (3)
N1—C1	1.402 (2)	C8—H8	0.9300
N1—C7	1.448 (3)	C6—H6A	0.9600
O1—C1	1.232 (3)	C6—H6B	0.9600
C1—C2	1.432 (3)	C6—H6C	0.9600
C5—C4	1.349 (3)	C11—C10	1.375 (4)
C5—H5	0.9300	C11—H11	0.9300
C7—C8	1.378 (3)	C3—C2	1.338 (3)
C7—C12	1.384 (3)	C3—H3	0.9300
C4—C3	1.417 (3)	C2—H2	0.9300
C4—C6	1.501 (3)	C9—C10	1.376 (4)
C12—C11	1.386 (4)	C9—H9	0.9300
C12—H12	0.9300	C10—H10	0.9300
			
C5—N1—C1	121.93 (17)	C4—C6—H6A	109.5
C5—N1—C7	118.39 (16)	C4—C6—H6B	109.5
C1—N1—C7	119.67 (16)	H6A—C6—H6B	109.5
O1—C1—N1	120.88 (19)	C4—C6—H6C	109.5
O1—C1—C2	124.9 (2)	H6A—C6—H6C	109.5
N1—C1—C2	114.20 (18)	H6B—C6—H6C	109.5
C4—C5—N1	122.94 (19)	C10—C11—C12	120.7 (2)
C4—C5—H5	118.5	C10—C11—H11	119.7
N1—C5—H5	118.5	C12—C11—H11	119.7
C8—C7—C12	120.74 (19)	C2—C3—C4	121.7 (2)
C8—C7—N1	119.42 (17)	C2—C3—H3	119.1
C12—C7—N1	119.81 (19)	C4—C3—H3	119.1
C5—C4—C3	116.47 (19)	C3—C2—C1	122.6 (2)
C5—C4—C6	122.2 (2)	C3—C2—H2	118.7
C3—C4—C6	121.4 (2)	C1—C2—H2	118.7
C7—C12—C11	119.0 (2)	C10—C9—C8	120.6 (2)
C7—C12—H12	120.5	C10—C9—H9	119.7
C11—C12—H12	120.5	C8—C9—H9	119.7
C7—C8—C9	119.3 (2)	C11—C10—C9	119.7 (2)
C7—C8—H8	120.4	C11—C10—H10	120.2
C9—C8—H8	120.4	C9—C10—H10	120.2

### Hydrogen-bond geometry (Å, º)

**Table:**

*D*—H···*A*	*D*—H	H···*A*	*D*···*A*	*D*—H···*A*
C8—H8···O1^i^	0.93	2.33	3.203 (3)	156
C10—H10···O1^ii^	0.93	2.46	3.310 (3)	152

Symmetry codes: (i) *x*−1, *y*, *z*; (ii) −*x*+2, *y*−1/2, −*z*+2.
