# Supplementary material for: Crystal structure of pirfenidone (5-methyl-1-phenyl-1H-pyridin-2-one): an active pharmaceutical ingredient (API)
Source: Acta Crystallogr E Crystallogr Commun. 2019 Jun 11;75(Pt 7):984–6. doi: 10.1107/S2056989019006418 (PMC6659322; doi:10.1107/S2056989019006418)

Search Overview

Search:	search4
Date/Time done:	Sun May 19 12:50:34 2019
Database(s):	CSD version 5.40 updates (Feb 2019)<br>CSD version 5.40 (November 2018)
Restriction Info:	No refcode restrictions applied
Filters:	None
Percentage Completed:	100%
Number of Hits:	40

Single query used. Search found structures that:

match

Query 1

Query 1

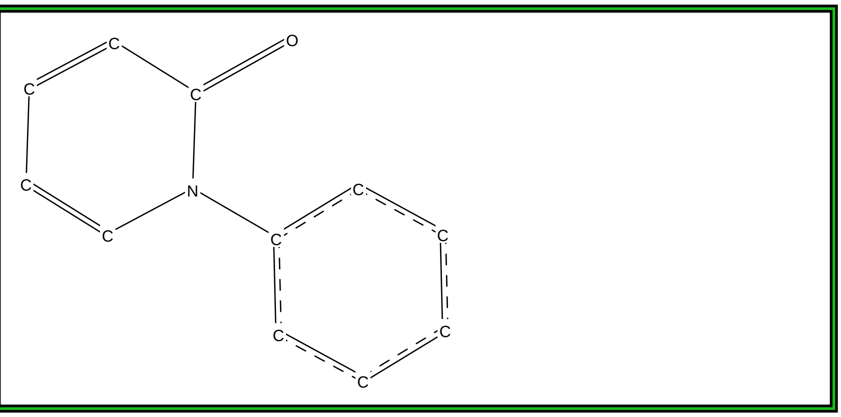

Search: search4 (Sun May 19 12:50:34 2019): Hits 1-4

AQIKIV

Reference: V.P.Tkachova, N.Yu.Gorobets, R.P.Tkachov, O.D.Dyachenko, E.B.Rusanov, V.D.Dyachenko (2010) *ARKIVOC* ,**11,** 254-11

Formula: $C_{19} H_{14} N_4 O_2$

Compound Name: 2-Amino-5-cyano-6-oxo-N,1-diphenyl-1,6-dihydropyridine-3-carboxamide

Space Group:	P21	***Cell:***	***a*** 9.216(1)	***b*** 8.239(1)	***c*** 10.873(1)
Space Group No.:	4	***(Å,°)***	α 90.00	β 93.05(0)	γ 90.00

R-Factor (%): 4.20 ***Temperature(K):*** 296 ***Density(g/cm³):*** 1.331

Parameters

Fragment 1
ANG1 (A) 73.016

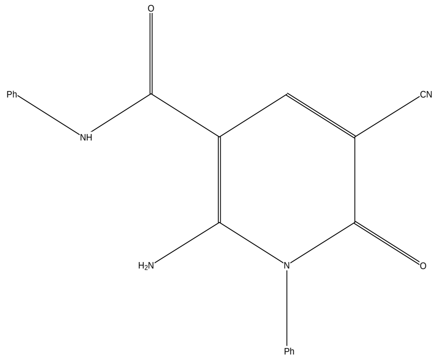

AZANOE

Reference: G.H.Elgemeie, P.G.Jones (2004) *Acta Crystallogr.,Sect.E:Struct.Rep.Online* ,**60,**o2107

Formula: $C_{15} H_{12} N_4 O_1 S_1$

Compound Name: 6-Amino-4-(methylsulfanyl)-2-oxo-1-tolyl-1,2-dihydropyridine-3,5-dicarbonitrile

Space Group:	P21/n	***Cell:***	***a*** 8.560(0)	***b*** 9.611(0)	***c*** 17.932(0)
Space Group No.:	14	***(Å,°)***	α 90.00	β 100.36(0)	γ 90.00

R-Factor (%): 3.28 ***Temperature(K):*** 173 ***Density(g/cm³):*** 1.356

Parameters

Fragment 1
ANG1 (A) 85.557

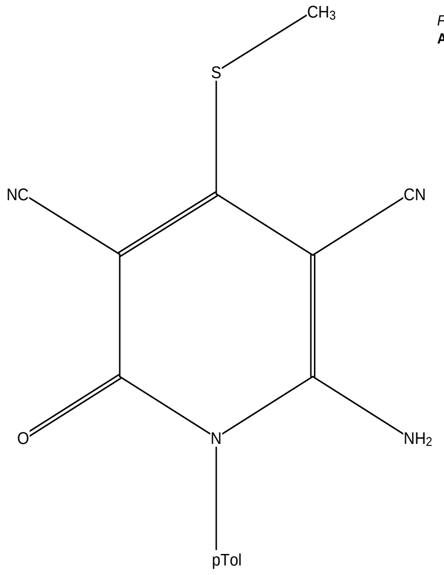

BAFPUV

Reference: V.D.Dyachenko, O.S.Butyukova, A.D.Dyachenko, O.V.Shishkin (2011) *Zh.Obshch.Khim.(Russ.)(Russ.J.Gen.Chem.)* ,**81,** 857

Formula: $C_{21} H_{18} N_4 O_3 S_1$

Compound Name: (rac)-2-Amino-5-cyano-N-(2-methoxyphenyl)-4-(methylsulfanyl)-6-oxo-1-phenyl-1,6-dihydropyridine-3-carboxamide

Space Group:	Pca21	***Cell:***	***a*** 35.753(1)	***b*** 5.464(0)	***c*** 9.937(0)
Space Group No.:	29	***(Å,°)***	α 90.00	β 90.00	γ 90.00

R-Factor (%): 4.58 ***Temperature(K):*** 295 ***Density(g/cm³):*** 1.391

Parameters

Fragment 1
ANG1 (A) 84.616

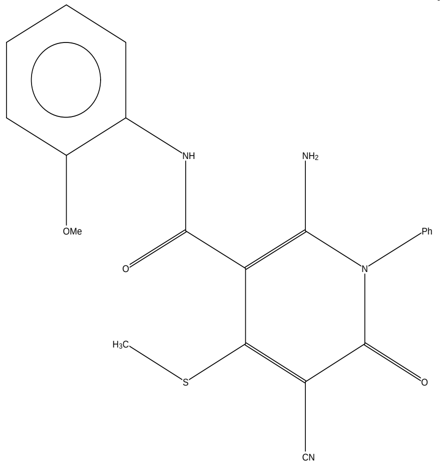

BAFQAC

Reference: V.D.Dyachenko, O.S.Butyukova, A.D.Dyachenko, O.V.Shishkin (2011) *Zh.Obshch.Khim.(Russ.)(Russ.J.Gen.Chem.)* ,**81,** 857

Formula: $C_{21} H_{18} N_4 O_3 S_1$

Compound Name: 2-Amino-5-cyano-1-(2-methoxyphenyl)-4-(methylsulfanyl)-6-oxo-N-phenyl-1,6-dihydropyridine-3-carboxamide

Space Group:	P21/c	***Cell:***	***a*** 9.189(1)	***b*** 16.664(3)	***c*** 13.221(3)
Space Group No.:	14	***(Å,°)***	α 90.00	β 98.78(1)	γ 90.00

R-Factor (%): 5.68 ***Temperature(K):*** 295 ***Density(g/cm³):*** 1.349

Parameters

Fragment 1
ANG1 (A) 80.908

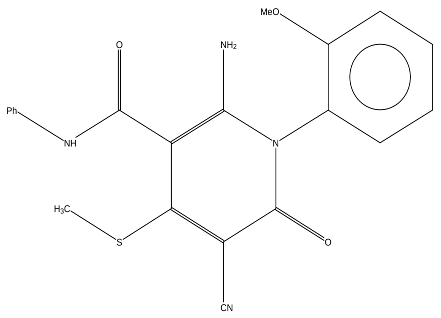

Search: search4 (Sun May 19 12:50:34 2019): Hits 5-8

BAMTOB

Reference: S.S.Hayotsyan, A.H.Hasratyan, S.G.Konkova, A.Kh.Khachatryan, A.E.Badasyan, A.G.Ayvazyan, G.A.Panosyan, M.S.Sargsyan (2014) *Chemistry of Heterocyclic Compounds* ,**50,**1126

Formula: C_{19} H_{21} N_1 O_4

Compound Name: ethyl 5-acetyl-1-(2,4-dimethylphenyl)-6-methyl-2-oxo-1,2-dihydropyridine-3-carboxylate

Space Group:	P-1	***Cell:***	***a*** 8.847(1)	***b*** 10.083(2)	***c*** 11.343(2)
Space Group No.:	2	***(Å,°)***	α 111.01(3)	β 96.31(3)	γ 107.42(3)

R-Factor (%): 5.43 ***Temperature(K):*** 293 ***Density(g/cm³):*** 1.244

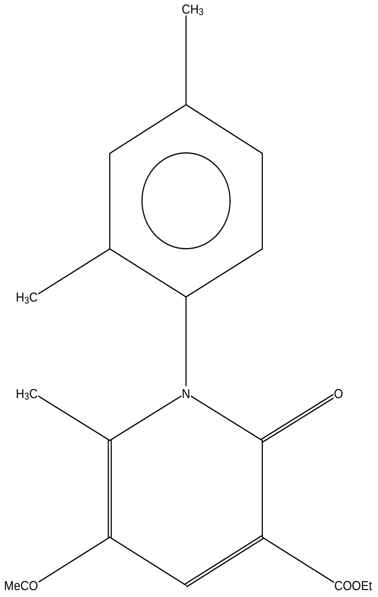

Parameters

Fragment 1
ANG1 (A) 85.446

DACNIG

Reference: Xiuyun Yang, Yanyan Cheng, Fei Zhao, Yunhui Li, Yuanli Ding, Yongjiu Liang, Fengjiao Gan, Dewen Dong (2011) *Tetrahedron* ,**67,**8343

Formula: C_{19} H_{14} Cl_1 N_5 O_1 S_1

Compound Name: ((6-Amino-1-(4-chlorophenyl)-5-cyano-4-methyl-2-oxo-1,2-dihydropyridin-3-yl)(ethylsulfanyl)methylene)malononitrile

Space Group:	Pbca	***Cell:***	***a*** 16.362(1)	***b*** 10.661(1)	***c*** 22.235(2)
Space Group No.:	61	***(Å,°)***	α 90.00	β 90.00	γ 90.00

R-Factor (%): 4.47 ***Temperature(K):*** 298 ***Density(g/cm³):*** 1.356

Parameters

Fragment 1
ANG1 (A) 76.040

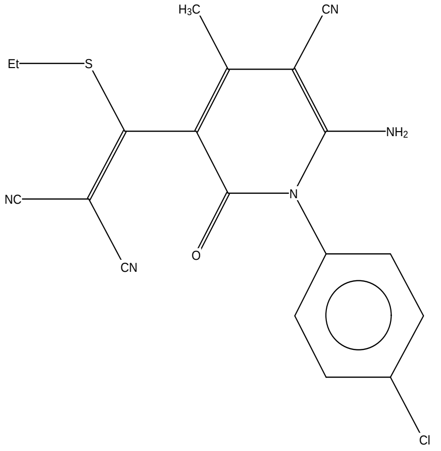

DITSOQ

Reference: K.Keshav, A.J.Elias (2013) *Inorg.Chem.Commun.* ,**35,**346

Formula: C_{25} H_{23} Fe_1 N_1 O_4

Compound Name: (2-(Ethoxycarbonyl)-4-(4-ethylphenyl)-3-oxocyclohexa-1,5-dien-1-yl) ferrocene

Space Group:	P-1	***Cell:***	***a*** 7.249(1)	***b*** 10.326(1)	***c*** 15.293(2)
Space Group No.:	2	***(Å,°)***	α 97.17(0)	β 103.27(0)	γ 102.86(0)

R-Factor (%): 4.09 ***Temperature(K):*** 293 ***Density(g/cm³):*** 1.423

Parameters

Fragment 1
ANG1 (A) 62.153

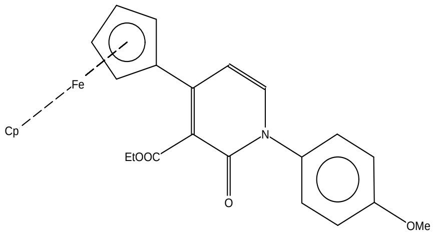

DOCKIP

Reference: M.J.Arevalo, M.Avalos, R.Babiano, P.Cintas, M.B.Hursthouse, J.L.Jimenez, M.E.Light, I.Lopez, J.C.Palacios (2000) *Tetrahedron* ,**56,**1247

Formula: C_{27} H_{23} N_3 O_5

Compound Name: 6-(Benzyl(methyl)amino)-5-methoxycarbonyl-1-(4-nitrophenyl)-3-phenylpyridin-2-one

Space Group:	P-1	***Cell:***	***a*** 10.097(0)	***b*** 10.387(0)	***c*** 13.135(0)
Space Group No.:	2	***(Å,°)***	α 99.62(0)	β 101.52(0)	γ 116.23(0)

R-Factor (%): 4.39 ***Temperature(K):*** 150 ***Density(g/cm³):*** 1.346

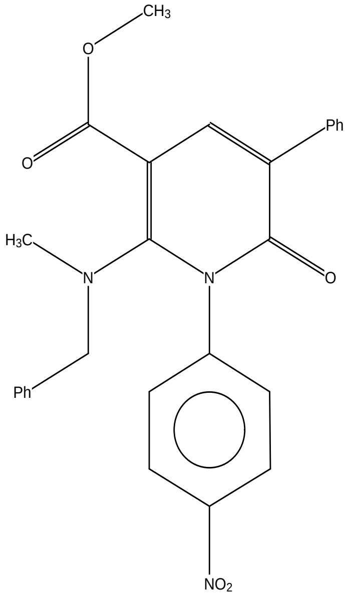

Parameters

Fragment 1
ANG1 (A) 71.977

Search: search4 (Sun May 19 12:50:34 2019): Hits 9-12

EBATIK

Reference: M.Kozul, Z.Stiplosek, Z.Orhanovic, K.Jakopcic, A.Nagl, A.Hergold-Brundic (1999) *J.Heterocycl.Chem.* ,**36,**493

Formula: $C_{27} H_{25} N_3 O_5,0.5(C_1 H_4 O_1)$

Compound Name: 5-Hydroxy-3-(4-methoxyphenylamino)-6-(4-methoxyphenylimino)methyl-N-(4-methoxyphenyl)-2-pyridone methanol solvate

Space Group: C2/c ***Cell:*** ***a*** 16.046(5) ***b*** 8.755(3) ***c*** 36.790(9)
Space Group No.: 15 ***(Å,°)*** α 90.00 β 97.42(2) γ 90.00

R-Factor (%): 7.14 ***Temperature(K):*** 295 ***Density(g/cm3):*** 1.264

Parameters

Fragment 1
ANG1 (A) 85.493

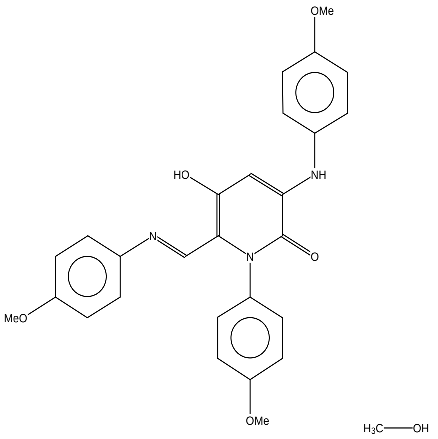

FEQJET

Reference: P.Manchanda, B.Parshad, A.Kumar, R.Tiwari, A.Shirazi, K.Parang, S.Sharma (2017) *Arch.Pharm.Chem.Life Sci.* ,**350,**e1600390

Formula: $C_{28} H_{20} F_1 N_5 O_3,x(C_1 H_1 Cl_3)$

Compound Name: 5-(4-fluoro-2-hydroxybenzoyl)-1-(4-methyl-3-((4-(pyridin-3-yl)pyrimidin-2-yl)amino)phenyl)pyridin-2(1H)-one chloroform solvate

Space Group: P21 ***Cell:*** ***a*** 10.417(5) ***b*** 21.881(5) ***c*** 14.589(5)
Space Group No.: 4 ***(Å,°)*** α 90.00 β 104.23(0) γ 90.00

R-Factor (%): 11.36 ***Temperature(K):*** 173 ***Density(g/cm3):*** 1.263

Parameters

Fragment 1
ANG1 (A) 64.446

Fragment 2
ANG1 (A) 72.722

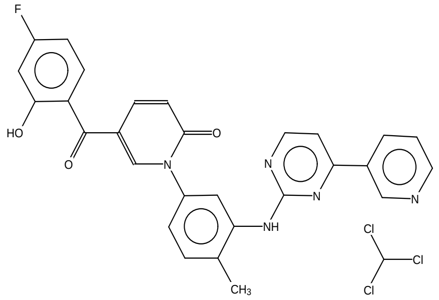

HANDIL

Reference: Jing Sun, Yan Sun, Er-Yan Xia, Chao-Guo Yan (2011) *ACS Comb. Sci.* ,**13,**436

Formula: $C_{21} H_{15} Cl_1 N_2 O_3$

Compound Name: Methyl 4-(4-chlorophenyl)-5-cyano-1-(4-methylphenyl)-6-oxo-1,6-dihydropyridine-3-carboxylate

Space Group: P21/c ***Cell:*** ***a*** 11.362(3) ***b*** 13.771(3) ***c*** 11.908(3)
Space Group No.: 14 ***(Å,°)*** α 90.00 β 95.54(0) γ 90.00

R-Factor (%): 4.19 ***Temperature(K):*** 296 ***Density(g/cm3):*** 1.357

Parameters

Fragment 1
ANG1 (A) 69.415

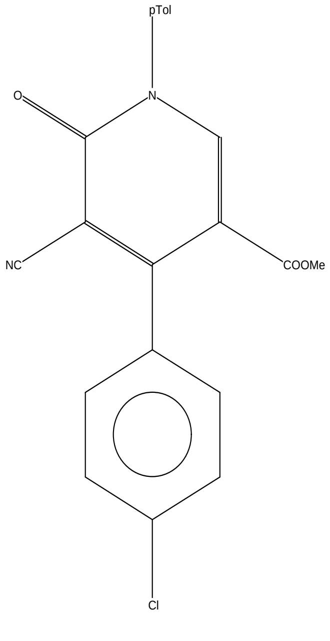

HURMUD

Reference: J.S.Siddle, A.S.Batsanov, S.T.Caldwell, G.Cooke, M.R.Bryce (2010) *Tetrahedron* ,**66,**6138

Formula: $C_{19} H_{17} N_1 O_3$

Compound Name: 1,5-bis(4-methoxyphenyl)pyridin-2(1H)-one

Space Group: P21/c ***Cell:*** ***a*** 10.647(0) ***b*** 12.516(0) ***c*** 11.497(0)
Space Group No.: 14 ***(Å,°)*** α 90.00 β 93.90(0) γ 90.00

R-Factor (%): 4.91 ***Temperature(K):*** 120 ***Density(g/cm3):*** 1.336

Parameters

Fragment 1
ANG1 (A) 68.568

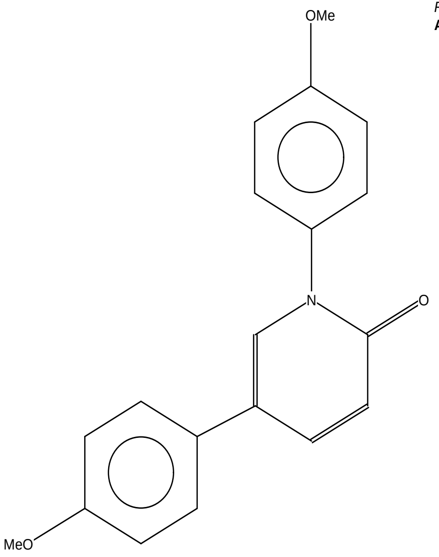

Search: search4 (Sun May 19 12:50:34 2019): Hits 13-16

IDOMAR

Reference: F.Ren, G.Li, Q.Zhang, J.Yao, X.Zhang (2013) *Acta Crystallogr.,Sect.E:Struct.Rep.Online* ,**69,**o732

Formula: $C_{18}\,H_{12}\,Cl_1\,N_1\,O_4$

Compound Name: 1-(3-Chlorophenyl)-5-(2,4-dihydroxybenzoyl)pyridin-2(1H)-one

Space Group:	P-1	***Cell:***	***a*** 6.689(3)	***b*** 9.009(4)	***c*** 13.257(6)		
Space Group No.:	2	***(Å,°)***	α 87.19(0)	β 87.72(0)	γ 82.67(0)		
R-Factor (%):	7.80	***Temperature(K):***	293	***Density(g/cm³):***	1.435		

Parameters

Fragment 1
ANG1 (A) 85.808

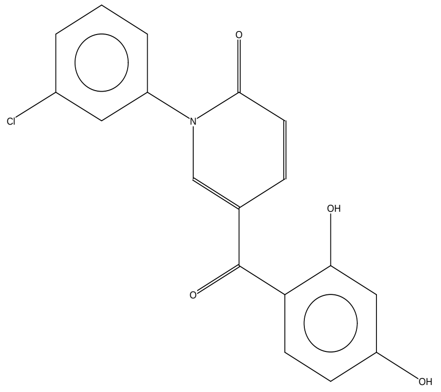

IQOREM

Reference: H.Reinke, I.O.Martinez, K.Peseke (2010) *CSD Communication(Private Communication) ,*

Formula: $C_{30}\,H_{33}\,N_1\,O_7$

Compound Name: 3-Acetyl-1,2-dihydro-1-(4-methoxyphenyl)-6-(methyl 2-O-benzyl-4,6-O-benzylidene-3-deoxy-α-D-altropyranosid-3-ylmethyl)-4-phenyl-pyridin-2-one

Synonym: 3-Acetyl-1-(4-methoxyphenyl)-4-phenyl-6-(2,2,2',2'-tetramethyl-4,4'-bi-1,3-dioxol-5-yl)pyridin-2(1H)-one

Space Group:	P21	***Cell:***	***a*** 12.165(0)	***b*** 9.001(0)	***c*** 12.224(0)
Space Group No.:	4	***(Å,°)***	α 90.00	β 90.34(0)	γ 90.00
R-Factor (%):	3.84	***Temperature(K):***	173	***Density(g/cm³):***	1.289

Parameters

Fragment 1
ANG1 (A) 86.707

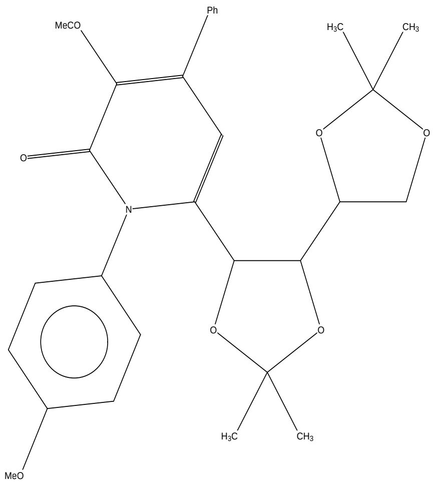

LIHKAP

Reference: Wei Pan, Dewen Dong, Kewei Wang, Jie Zhang, Rigenhada Wu, Dexuan Xiang, Qun Liu (2007) *Org.Lett.* ,**9,**2421

Formula: $C_{13}\,H_{10}\,Cl_3\,N_1\,O_1$

Compound Name: 4-Chloro-3-(2-chloroethyl)-1-(4-chlorophenyl)pyridin-2(1H)-one

Space Group:	P21/n	***Cell:***	***a*** 11.077(0)	***b*** 7.208(0)	***c*** 17.355(0)
Space Group No.:	14	***(Å,°)***	α 90.00	β 105.98(0)	γ 90.00
R-Factor (%):	4.18	***Temperature(K):***	293	***Density(g/cm³):***	1.509

Parameters

Fragment 1
ANG1 (A) 51.687

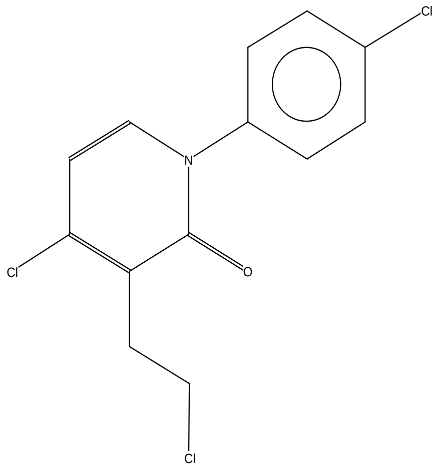

MAYWUF

Reference: H.Schirok, C.Alonso-Alija, J.Benet-Buchholz, A.H.Goller, R.Grosser, M.Michels, H.Paulsen (2005) *J.Org.Chem.* ,**70,**9463

Formula: $C_{20}\,H_{17}\,F_1\,N_2\,O_4,C_1\,H_1\,Cl_3$

Compound Name: (S)-6-Amino-5-(4-fluorobenzoyl)-1-(2,4-dimethoxyphenyl)-2(1H)-pyridone chloroform solvate

Space Group:	P21	***Cell:***	***a*** 11.610(0)	***b*** 7.543(0)	***c*** 12.852(0)
Space Group No.:	4	***(Å,°)***	α 90.00	β 104.62(0)	γ 90.00
R-Factor (%):	5.48	***Temperature(K):***	90	***Density(g/cm³):***	1.487

Parameters

Fragment 1
ANG1 (A) 83.936

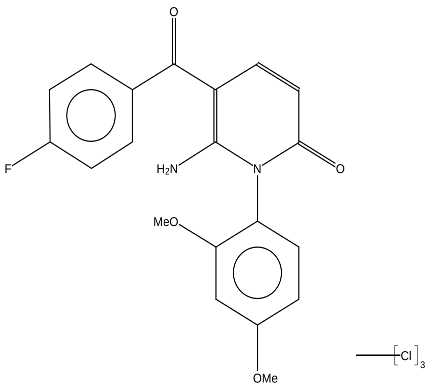

Search: search4 (Sun May 19 12:50:34 2019): Hits 17-20

MOMTIT

Reference: Wei Liu, Jing Yuan, Shi-Jun Zhang, Wei-Ren Xu, Chang-Jiang Huang, Li-Da Tang (2014) *Jiegou Huaxue(Chin.)(Chin.J.Struct.Chem.)* ,**33,**1091

Formula: $C_{20}\,H_{16}\,Cl_1\,N_3\,O_4\,S_1$

Compound Name: 5-chloro-N-((2-oxo-3-(4-(2-oxopyridin-1(2H)-yl)phenyl)-1,3-oxazolidin-5-yl)methyl)thiophene-2-carboxamide

Space Group:	P21	***Cell:***	***a***	5.790(1)	***b***	13.086(3)	***c***	12.889(3)
Space Group No.:	4	***(Å,°)***	α	90.00	β	100.86(3)	γ	90.00

R-Factor (%): 3.22 ***Temperature(K):*** 293 ***Density(g/cm³):*** 1.489

Parameters

Fragment 1
ANG1 (A) 56.071

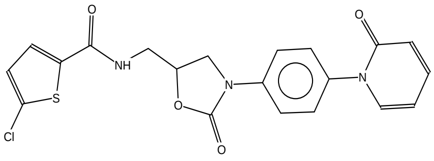

NAQXEL

Reference: R.S.G.R.Seixas, G.C.Ribeiro, S.Guieu, A.M.S.Silva (2016) *Chem. Sel.* ,**1,**318

Formula: $C_{20}\,H_{15}\,N_1\,O_6$

Compound Name: 5-(2-hydroxybenzoyl)-1-(4-methoxyphenyl)-2-oxo-1,2-dihydropyridine-3-carboxylic acid

Space Group:	P21/c	***Cell:***	***a***	8.359(0)	***b***	8.092(0)	***c***	24.902(1)
Space Group No.:	14	***(Å,°)***	α	90.00	β	95.45(0)	γ	90.00

R-Factor (%): 3.42 ***Temperature(K):*** 150 ***Density(g/cm³):*** 1.447

Parameters

Fragment 1
ANG1 (A) 61.777

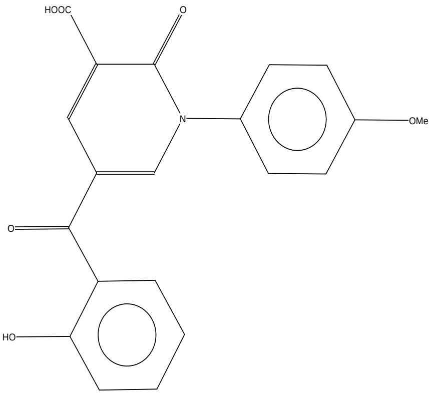

NEDWUO

Reference: V.Bertolasi, P.Gilli, V.Ferretti, G.Gilli (1998) *Acta Crystallogr.,Sect.B:Struct.Sci.* ,**54,**50

Formula: $C_{18}\,H_{14}\,Cl_2\,N_2\,O_1$

Compound Name: 1-(4-Chlorophenyl)-4-(4-chlorophenylamino)-6-methyl-2-pyridone

Space Group:	Pbca	***Cell:***	***a***	14.244(2)	***b***	9.645(1)	***c***	23.711(3)
Space Group No.:	61	***(Å,°)***	α	90.00	β	90.00	γ	90.00

R-Factor (%): 4.10 ***Temperature(K):*** 295 ***Density(g/cm³):*** 1.408

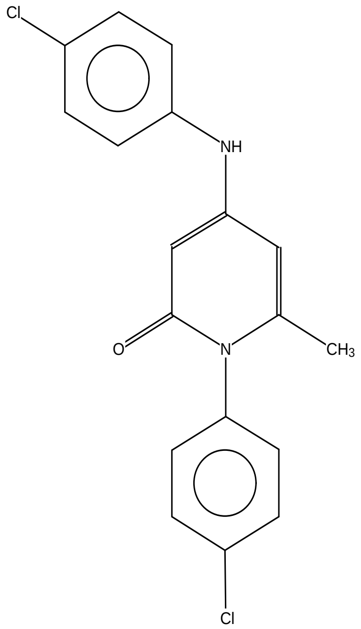

Parameters

Fragment 1
ANG1 (A) 76.625

NESBAO

Reference: G.A.Cartwright, R.O.Gould, H.McNab (1997) *Chem.Commun.* ,1293

Formula: $C_{15}\,H_{15}\,N_1\,O_2$

Compound Name: 5-Acetyl-N-p-tolyl-4-methyl-2-pyridone

Space Group:	P212121	***Cell:***	***a***	5.557(4)	***b***	9.645(4)	***c***	24.272(10)
Space Group No.:	19	***(Å,°)***	α	90.00	β	90.00	γ	90.00

R-Factor (%): 2.60 ***Temperature(K):*** 295 ***Density(g/cm³):*** 1.232

Parameters

Fragment 1
ANG1 (A) 81.481

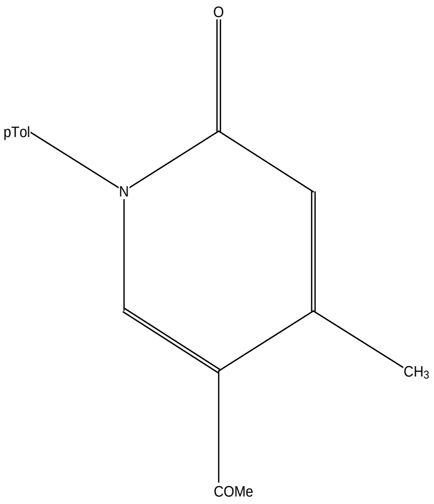

Search: search4 (Sun May 19 12:50:34 2019): Hits 21-24

NOLBIA

Reference: Jun Liu, Deqiang Liang, Mang Wang, Qun Liu (2008) *Synthesis* ,3633

Formula: $C_{14} H_{13} N_1 O_2 S_1$

Compound Name: S-Ethyl 2-oxo-1-phenyl-1,2-dihydro-3-pyridinecarbothioate

Space Group: P21
Space Group No.: 4

Cell: ***(Å,°)*** ***a*** 5.643(1) ***b*** 12.163(3) ***c*** 9.504(2) α 90.00 β 90.00 γ 90.00

R-Factor (%): 4.28 ***Temperature(K):*** 293 ***Density(g/cm^3):*** 1.320

Parameters

Fragment 1
ANG1 (A) 65.502

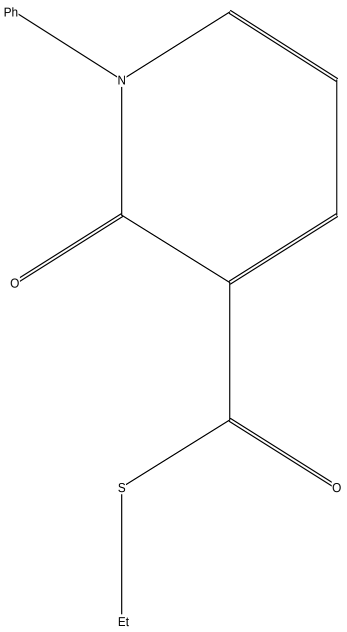

PAVQIO

Reference: M.S.Al-Said, M.M.Ghorab, H.A.Ghabbour, S.Arshad, H.-K.Fun (2012) *Acta Crystallogr.,Sect.E:Struct.Rep.Online* ,**68,**o1679

Formula: $C_{16} H_{16} N_2 O_1$

Compound Name: 1-(3-Ethylphenyl)-4,6-dimethyl-2-oxo-1,2-dihydropyridine-3-carbonitrile

Space Group: P21/c
Space Group No.: 14

Cell: ***(Å,°)*** ***a*** 8.383(0) ***b*** 7.185(0) ***c*** 23.526(0) α 90.00 β 93.20(0) γ 90.00

R-Factor (%): 4.74 ***Temperature(K):*** 296 ***Density(g/cm^3):*** 1.184

Parameters

Fragment 1
ANG1 (A) 85.331

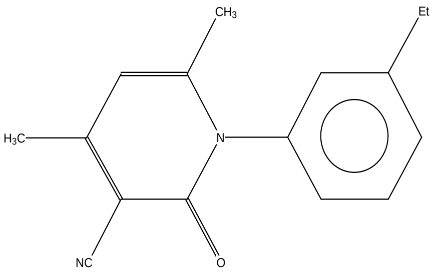

POSZEE

Reference: Qian Zhang, Xu Liu, Xiaoqing Xin, Rui Zhang, Yongjiu Liang, Dewen Dong (2014) *Chem.Commun.* ,**50,**15378

Formula: $C_{15} H_{16} N_2 O_3 S_1$

Compound Name: 3-Acetyl-6-amino-1-(4-methoxyphenyl)-5-(methylthio)pyridin-2(1H)-one

Space Group: Pccn
Space Group No.: 56

Cell: ***(Å,°)*** ***a*** 18.861(1) ***b*** 9.284(0) ***c*** 16.581(1) α 90.00 β 90.00 γ 90.00

R-Factor (%): 4.58 ***Temperature(K):*** 293 ***Density(g/cm^3):*** 1.393

Parameters

Fragment 1
ANG1 (A) 86.203

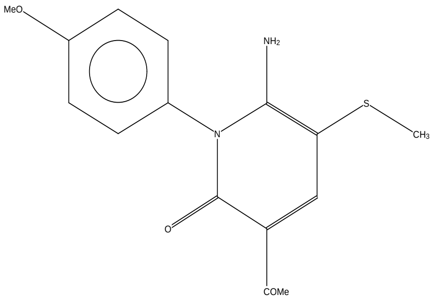

QERTEN

Reference: Chenguang Li, P.D.Robinson, D.J.Dyer (2006) *Acta Crystallogr.,Sect.E:Struct.Rep.Online* ,**62,**o5045

Formula: $C_{16} H_{12} N_2 O_2$

Compound Name: 1-(4-(2-Oxopyridin-1(2H)-yl)phenyl)pyridin-2(1H)-one

Space Group: P-1
Space Group No.: 2

Cell: ***(Å,°)*** ***a*** 5.891(1) ***b*** 6.703(1) ***c*** 8.871(1) α 76.50(3) β 71.33(3) γ 67.44(3)

R-Factor (%): 4.00 ***Temperature(K):*** 295 ***Density(g/cm^3):*** 1.444

Parameters

Fragment 1
ANG1 (A) 55.947

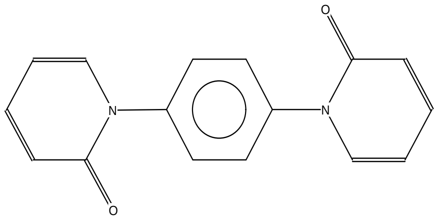

Search: search4 (Sun May 19 12:50:34 2019): Hits 25-28

QIWFIM

Reference: Dexuan Xiang, Kewei Wang, Yongjiu Liang, Guangyuan Zhou, Dewen Dong (2008) *Org.Lett.* ,**10,**345

Formula: $C_{12} H_8 Cl_1 N_1 O_2$

Compound Name: 4-Chloro-6-oxo-1-phenyl-1,6-dihydropyridine-3-carbaldehyde

Space Group: P21/c
Space Group No.: 14

Cell: ***(Å,°)*** ***a*** 11.067(5) ***b*** 12.890(5) ***c*** 7.536(5) α 90.00 β 103.02(0) γ 90.00

R-Factor (%): 3.23 ***Temperature(K):*** 293 ***Density(g/cm³):*** 1.482

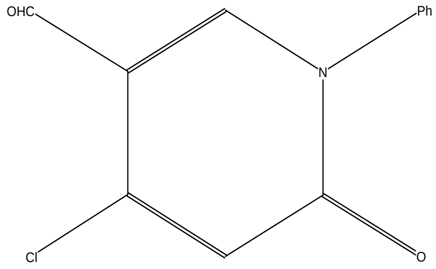

Parameters

Fragment 1
ANG1 (A) 64.659

SAPTUA

Reference: E.Kumarasamy, J.L.Jesuraj, J.N.Omlid, A.Ugrinov, J.Sivaguru (2011) *J.Am.Chem.Soc.* ,**133,**17106

Formula: $C_{15} H_{17} N_1 O_1$

Compound Name: (M)-(-)-1-(2-t-Butylphenyl)pyridin-2(1H)-one

Space Group: P21
Space Group No.: 4

Cell: ***(Å,°)*** ***a*** 15.318(0) ***b*** 11.433(0) ***c*** 16.002(0) α 90.00 β 117.60(0) γ 90.00

R-Factor (%): 4.19 ***Temperature(K):*** 100 ***Density(g/cm³):*** 1.216

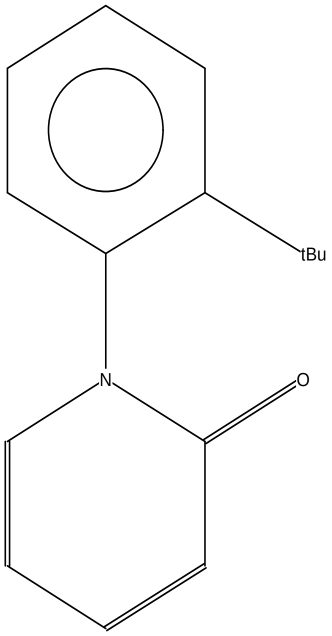

Parameters

Fragment 1
ANG1 (A) 85.488

Fragment 2
ANG1 (A) 85.583

Fragment 3
ANG1 (A) 87.992

Fragment 4
ANG1 (A) 89.480

SAPVAI

Reference: E.Kumarasamy, J.L.Jesuraj, J.N.Omlid, A.Ugrinov, J.Sivaguru (2011) *J.Am.Chem.Soc.* ,**133,**17106

Formula: $C_{15} H_{17} N_1 O_1$

Compound Name: (P)-(+)-1-(2-t-Butylphenyl)pyridin-2(1H)-one

Space Group: P21
Space Group No.: 4

Cell: ***(Å,°)*** ***a*** 15.322(0) ***b*** 11.427(0) ***c*** 16.041(0) α 90.00 β 117.82(0) γ 90.00

R-Factor (%): 6.87 ***Temperature(K):*** 100 ***Density(g/cm³):*** 1.216

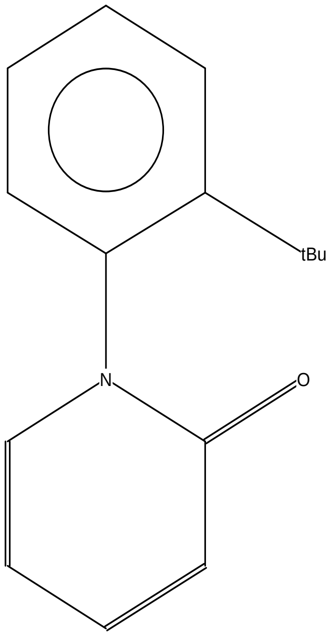

Parameters

Fragment 1
ANG1 (A) 85.456

Fragment 2
ANG1 (A) 85.825

Fragment 3
ANG1 (A) 88.193

Fragment 4
ANG1 (A) 89.578

SAPVAI01

Reference: E.Kumarasamy, J.L.Jesuraj, J.N.Omlid, A.Ugrinov, J.Sivaguru (2011) *J.Am.Chem.Soc.* ,**133,**17106

Formula: $C_{15} H_{17} N_1 O_1$

Compound Name: (P)-(+)-1-(2-t-Butylphenyl)pyridin-2(1H)-one

Space Group: P212121
Space Group No.: 19

Cell: ***(Å,°)*** ***a*** 6.875(0) ***b*** 12.488(0) ***c*** 30.403(0) α 90.00 β 90.00 γ 90.00

R-Factor (%): 2.61 ***Temperature(K):*** 100 ***Density(g/cm³):*** 1.157

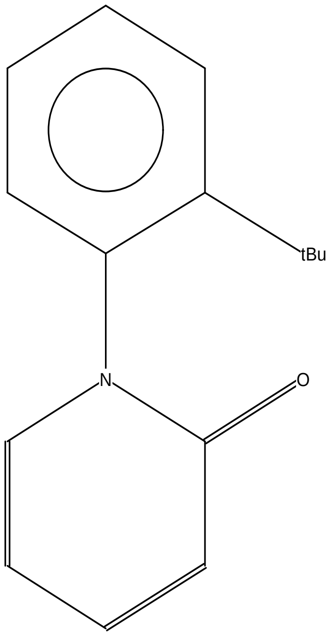

Parameters

Fragment 1
ANG1 (A) 84.911

Fragment 2
ANG1 (A) 86.448

Search: search4 (Sun May 19 12:50:34 2019): Hits 29-32

SAPVIQ

Reference: E.Kumarasamy, J.L.Jesuraj, J.N.Omlid, A.Ugrinov, J.Sivaguru (2011) *J.Am.Chem.Soc.* ,**133,**17106

Formula: $C_{14} H_{15} N_1 O_2$

Compound Name: 1-(2-(2-Hydroxypropan-2-yl)phenyl)pyridin-2(1H)-one

Space Group:	Pbca	***Cell:***	***a***	8.577(0)	***b***	15.017(0)	***c***	18.426(0)
Space Group No.:	61	***(Å,°)***	α	90.00	β	90.00	γ	90.00

R-Factor (%): 3.69 ***Temperature(K):*** 100 ***Density(g/cm^3):*** 1.283

Parameters

Fragment 1
ANG1 (A) 86.955

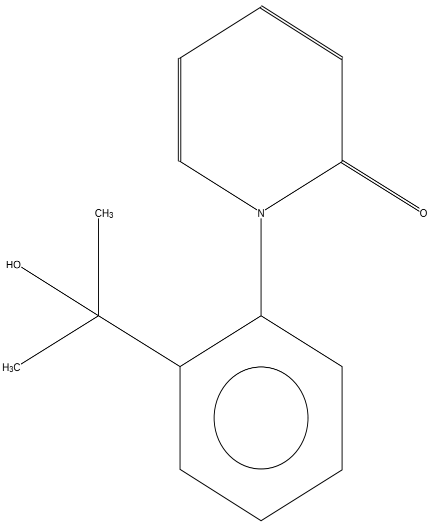

SAPVOW

Reference: E.Kumarasamy, J.L.Jesuraj, J.N.Omlid, A.Ugrinov, J.Sivaguru (2011) *J.Am.Chem.Soc.* ,**133,**17106

Formula: $C_{24} H_{19} N_1 O_2$

Compound Name: 1-(2-(Hydroxy(diphenyl)methyl)phenyl)pyridin-2(1H)-one

Space Group:	P21/n	***Cell:***	***a***	17.601(10)	***b***	6.388(4)	***c***	18.243(10)
Space Group No.:	14	***(Å,°)***	α	90.00	β	114.99(0)	γ	90.00

R-Factor (%): 4.68 ***Temperature(K):*** 293 ***Density(g/cm^3):*** 1.263

Parameters

Fragment 1
ANG1 (A) 76.262

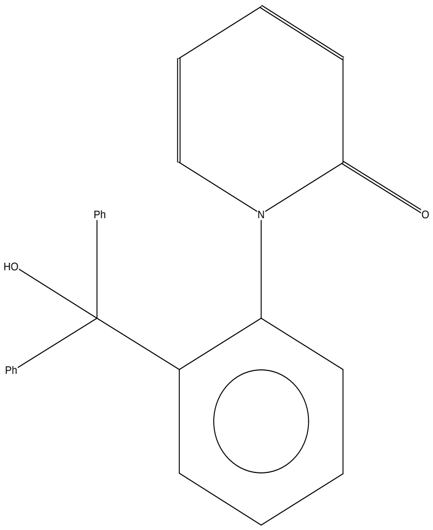

TEMKIH

Reference: Yushang Shao, Wenjun Yao, Jun Liu, Kai Zhu, Yanzhong Li (2012) *Synthesis* ,**44,**3301

Formula: $C_{20} H_{15} N_1 O_4$

Compound Name: Methyl 5-benzoyl-2-oxo-1-phenyl-1,2-dihydropyridine-4-carboxylate

Space Group:	Pna21	***Cell:***	***a***	20.251(1)	***b***	10.933(0)	***c***	14.994(1)
Space Group No.:	33	***(Å,°)***	α	90.00	β	90.00	γ	90.00

R-Factor (%): 6.71 ***Temperature(K):*** 293 ***Density(g/cm^3):*** 1.334

Parameters

Fragment 1
ANG1 (A) 55.829

Fragment 2
ANG1 (A) 57.123

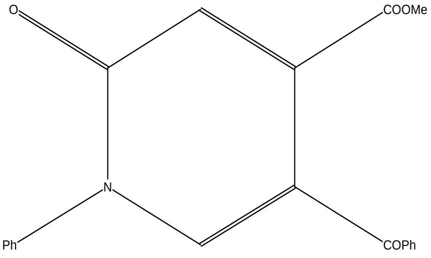

USOTIH

Reference: M.Hebert, P.Petiot, E.Benoit, J.Dansereau, T.Ahmad, A.Le Roch, X.Ottenwaelder, A.Gagnon (2016) *J.Org.Chem.* ,**81,**5401

Formula: $C_{17} H_{14} N_2 O_3$

Compound Name: ethyl 3-(3-(3-cyano-2-oxopyridin-1(2H)-yl)phenyl)acrylate

Space Group:	P-1	***Cell:***	***a***	7.015(0)	***b***	7.074(0)	***c***	15.121(0)
Space Group No.:	2	***(Å,°)***	α	83.64(0)	β	80.15(0)	γ	73.30(0)

R-Factor (%): 4.72 ***Temperature(K):*** 150 ***Density(g/cm^3):*** 1.383

Parameters

Fragment 1
ANG1 (A) 57.137

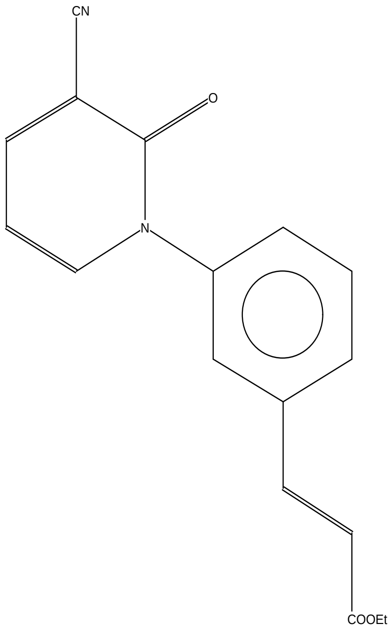

Search: search4 (Sun May 19 12:50:34 2019): Hits 33-36

VATBOJ

Reference: Li Xing, B.Devadas, R.V.Devraj, S.R.Selness, Huey Shieh, J.K.Walker, M.Mao, D.Messing, B.Samas, J.Z.Yang, G.D.Anderson, E.G.Webb, J.B.Monahan (2012) *ChemMedChem* ,**7,**273

Formula: $C_{22} H_{19} Br_1 F_2 N_2 O_3$

Compound Name: 3-(3-Bromo-4-((2,4-difluorobenzyl)oxy)-6-methyl-2-oxopyridin-1(2H)-yl)-N, 4-dimethylbenzamide

Synonym: PDB Chemical Component code: I45

Space Group:	P21	***Cell:***	***a*** 11.227(1)	***b*** 7.432(1)	***c*** 12.747(2)
Space Group No.:	4	***(Å,°)***	α 90.00	β 101.64(0)	γ 90.00

R-Factor (%): 4.82 ***Temperature(K):*** 301 ***Density(g/cm³):*** 1.522

Parameters

Fragment 1
ANG1 (A) 80.042

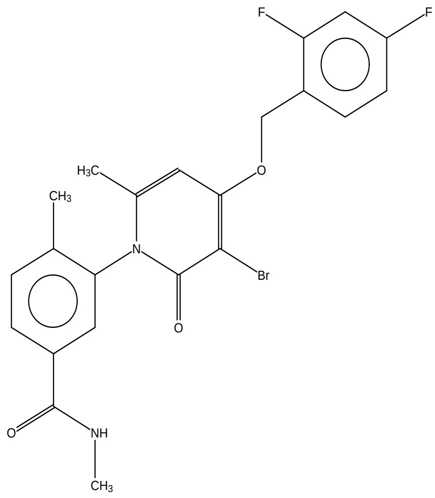

VIDNOM

Reference: Jin-Ping Xue, Hua-Li Zhu, Nai-Sheng Chen, Jin-Ling Huang (2007) *Acta Crystallogr.,Sect.E:Struct.Rep.Online* ,**63,** o2434

Formula: $C_{13} H_7 N_3 O_1$

Compound Name: 3-(2-Oxo-2H-pyridin-1-yl)phthalonitrile

Space Group:	P21/n	***Cell:***	***a*** 8.132(6)	***b*** 11.739(6)	***c*** 11.224(6)
Space Group No.:	14	***(Å,°)***	α 90.00	β 97.21(3)	γ 90.00

R-Factor (%): 3.96 ***Temperature(K):*** 173 ***Density(g/cm³):*** 1.382

Parameters

Fragment 1
ANG1 (A) 65.936

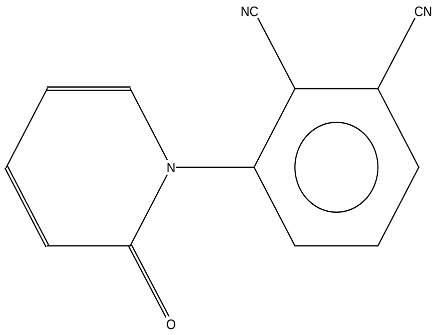

VISLUF

Reference: Li Chen, Yu-Long Zhao, Qun Liu, Chao Cheng, Cheng-Ri Piao (2007) *J.Org.Chem.* ,**72,**9259

Formula: $C_{15} H_{12} Cl_3 N_1 O_1 S_2$

Compound Name: 3-Chloropropyl 4-chloro-1-(4-chlorophenyl)-2-oxo-1,2-dihydropyridine-3-carbodithioate

Space Group:	P21/c	***Cell:***	***a*** 8.775(0)	***b*** 22.221(2)	***c*** 8.826(0)
Space Group No.:	14	***(Å,°)***	α 90.00	β 97.23(0)	γ 90.00

R-Factor (%): 3.83 ***Temperature(K):*** 293 ***Density(g/cm³):*** 1.528

Parameters

Fragment 1
ANG1 (A) 58.537

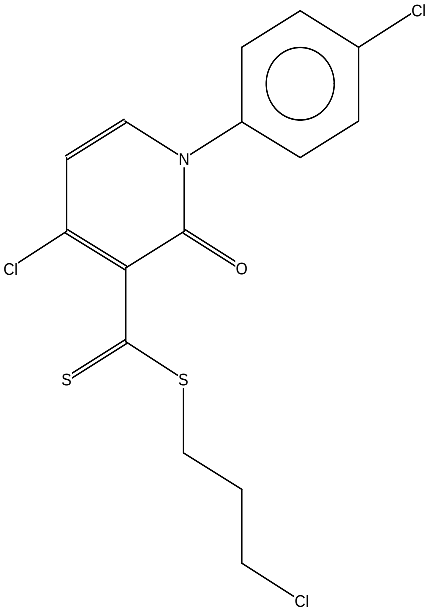

WEDCEP

Reference: C.Allais, O.Basle, J.-M.Grassot, M.Fontaine, S.Anguille, J.Rodriguez, T.Constantieux (2012) *Adv.Synth.Catal.* ,**354,**2084

Formula: $C_{20} H_{17} N_1 O_2$

Compound Name: 3-Acetyl-6-methyl-1,4-diphenylpyridin-2(1H)-one

Space Group:	P-1	***Cell:***	***a*** 7.413(0)	***b*** 16.092(0)	***c*** 21.111(0)
Space Group No.:	2	***(Å,°)***	α 102.31(0)	β 98.76(0)	γ 91.70(0)

R-Factor (%): 7.62 ***Temperature(K):*** 293 ***Density(g/cm³):*** 1.246

Parameters

Fragment 1
ANG1 (A) 82.032

Fragment 2
ANG1 (A) 84.090

Fragment 3
ANG1 (A) 89.280

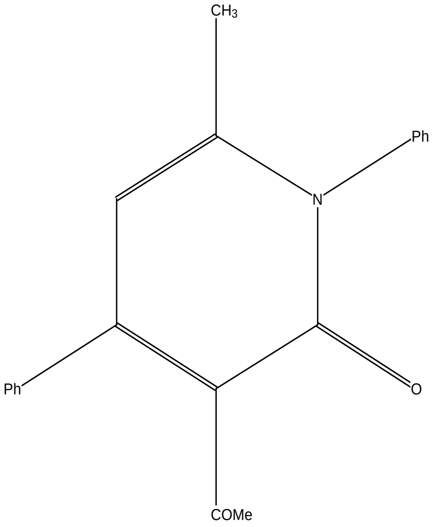

Search: search4 (Sun May 19 12:50:34 2019): Hits 37-40

XIQGAF

Reference: D.K.Rayabarapu, K.K.Majumdar, T.Sambaiah, Chein-Hong Cheng (2001) *J.Org.Chem.* ,**66,**3646

Formula: $C_{13}\,H_{11}\,N_1\,O_3$

Compound Name: methyl 2-(2-oxo-1,2-dihydro-1-pyridyl)benzoate

Space Group: P21/c ***Cell:*** ***a*** 9.362(1) ***b*** 12.970(1) ***c*** 9.287(1)
Space Group No.: 14 ***(Å,°)*** α 90.00 β 91.07(0) γ 90.00

R-Factor (%): 4.82 ***Temperature(K):*** 294 ***Density(g/cm³):*** 1.351

Parameters

Fragment 1
ANG1 (A) 86.445

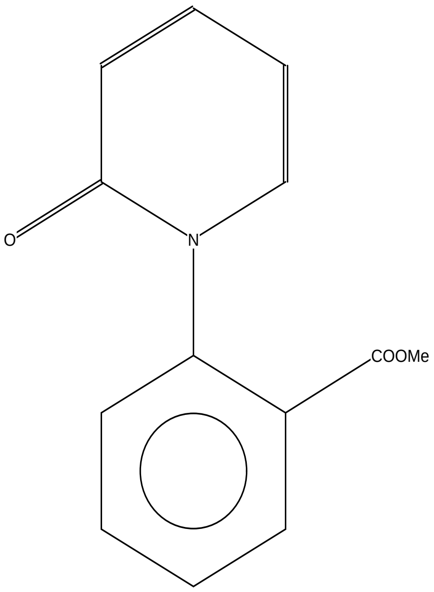

XIQGEJ

Reference: D.K.Rayabarapu, K.K.Majumdar, T.Sambaiah, Chein-Hong Cheng (2001) *J.Org.Chem.* ,**66,**3646

Formula: $C_{14}\,H_{13}\,N_1\,O_2$

Compound Name: 1-(2-acetyl-3-methylphenyl)-1,2-dihydro-2-pyridone

Space Group: P21/c ***Cell:*** ***a*** 8.171(0) ***b*** 7.945(0) ***c*** 19.026(2)
Space Group No.: 14 ***(Å,°)*** α 90.00 β 102.40(0) γ 90.00

R-Factor (%): 5.27 ***Temperature(K):*** 294 ***Density(g/cm³):*** 1.251

Parameters

Fragment 1
ANG1 (A) 84.265

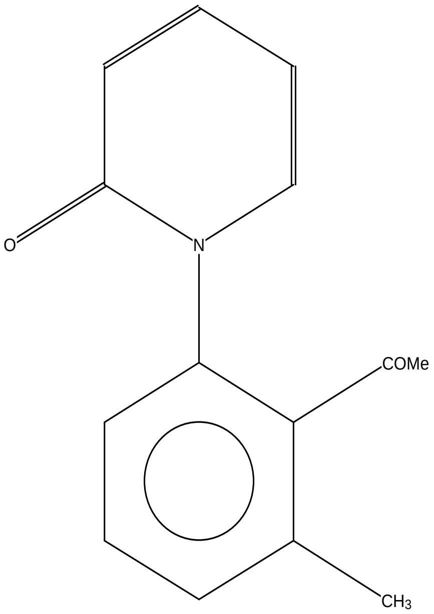

YAPBUO

Reference: T.Sengupta, K.S.Gayen, P.Pandit, D.K.Maiti (2012) *Chem.-Eur.J.* ,**18,**1905

Formula: $C_{22}\,H_{19}\,N_1\,O_4$

Compound Name: 3-Acetyl-5-(2-hydroxy-5-methylbenzoyl)-1-(4-methylphenyl)pyridin-2(1H)-one

Space Group: Pbca ***Cell:*** ***a*** 20.885(6) ***b*** 7.102(1) ***c*** 24.625(7)
Space Group No.: 61 ***(Å,°)*** α 90.00 β 90.00 γ 90.00

R-Factor (%): 4.52 ***Temperature(K):*** 296 ***Density(g/cm³):*** 1.314

Parameters

Fragment 1
ANG1 (A) 62.220

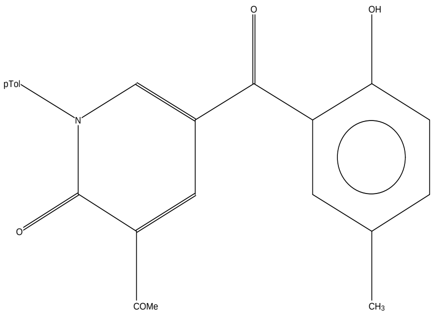

ZESVIF

Reference: Qing-Hu Teng, Xiang-Jun Peng, Zu-Yu Mo, Yan-Li Xu, Hai-Tao Tang, Heng-Shan Wang, Hong-Bin Sun, Ying-Ming Pan (2018) *Green Chemistry* ,**20,**2007

Formula: $C_{24}\,H_{19}\,N_1\,O_1$

Compound Name: 1-(4-methylphenyl)-4,6-diphenylpyridin-2(1H)-one

Space Group: P-1 ***Cell:*** ***a*** 9.462(0) ***b*** 9.753(0) ***c*** 10.539(0)
Space Group No.: 2 ***(Å,°)*** α 68.19(0) β 78.20(0) γ 88.78(0)

R-Factor (%): 4.21 ***Temperature(K):*** 150 ***Density(g/cm³):*** 1.270

Parameters

Fragment 1
ANG1 (A) 60.888

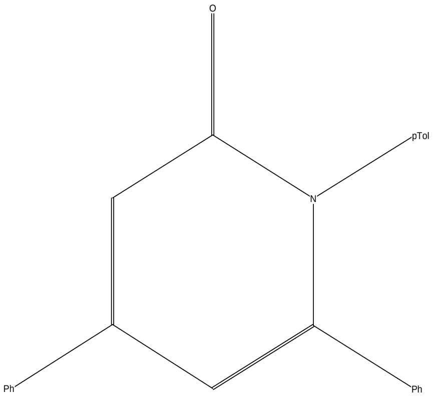

Supplement: Supplementary file 2 [file e-75-00984-sup3.pdf]
